# Non-Invasive Brain Stimulation to Improve Functional Recovery and Predict Outcome After Intracerebral Hemorrhage: A Narrative Review

**DOI:** 10.3390/jcm14020398

**Published:** 2025-01-10

**Authors:** Daniele Zanola, Andrea Morotti, Alessandro Padovani

**Affiliations:** 1 Neurology Unit, Department of Clinical and Experimental Sciences, University of Brescia, 25123 Brescia, Italy; d.zanola003@unibs.it (D.Z.);; 2 Department of Continuity of Care and Frailty, ASST Spedali Civili, 25123 Brescia, Italy

**Keywords:** intracerebral hemorrhage, prognosis, outcome, brain stimulation, TMS, recovery

## Abstract

Intracerebral hemorrhage (ICH) is a leading cause of stroke-related mortality and long-term disability, with initial ICH volume, age, location of the hemorrhage, and clinical severity being key predictors of outcome. While clinical scores incorporating these elements are validated and exhibit good inter-rater reliability, their accuracy in predicting long-term recovery remains suboptimal. Non-invasive brain stimulation (NIBS) has emerged as a potential adjunct for improving both prognostication and functional recovery in ICH survivors. Despite promising results, heterogeneity in stimulation protocols, patients’ populations, and outcome measures have prevented NIBS implementation in clinical practice. This narrative review summarizes the available evidence on the association between NIBS, outcome prediction and functional recovery, discussing current challenges and future perspectives.

## 1. Introduction

Intracerebral Hemorrhage (ICH) is a major determinant of death and disability worldwide, accounting for up to 50% of stroke-related mortality [1,2].

Baseline ICH volume, age, hemorrhage location, and clinical severity on admission, measured with the National Institutes of Health Stroke Scale (NIHSS) or Glasgow Coma Scale (GCS), are the main outcome predictors in ICH patients. Clinical scores incorporating these elements are validated and have shown a good inter-rater reliability, but their diagnostic performance remains suboptimal [3]. Furthermore, the majority of currently available prediction tools were designed to predict outcome at one or three months from the index event. However, the recovery trajectory of ICH is long, with room for functional outcome improvement up to one year [4,5]. Taken together, these elements suggest the need for novel prognostication tools and therapeutic strategies to improve long-term functional outcomes.

Non-Invasive Brain Stimulation (NIBS) has gained attention as a complementary approach to enhance both prognostication and functional recovery in stroke patients. However, inconsistencies in stimulation protocols, patient characteristics, and outcome assessments have hindered its adoption in routine clinical settings.

Previous studies on the application of NIBS in stroke patients focused primarily on ischemic stroke, whereas the use of these techniques remains less characterized in ICH survivors.

NIBS might provide an added value, improving ICH prognostication and promoting functional outcome improvement. The aim of this narrative review was to describe the available evidence supporting the potential use of NIBS to enhance functional recovery and improve the diagnostic accuracy of currently available tools for ICH prognostication.

## 2. Methods

We conducted a narrative review to explore the available evidence on the role of NIBS in functional recovery and outcome prognostication in acute ICH. All the studies were analyzed to provide general characteristics of included patients, focusing in particular on known predictors of outcome in ICH, as outlined in Table 1. Table 2 summarizes the main characteristics of included studies.

## 3. Overview of Nibs Methods

### 3.1. Transcranial Magnetic Stimulation (TMS)

A strong enough induced current from TMS can trigger neuronal depolarization, leading to an action potential [24]. Repetitive TMS (rTMS) delivers a series of pulses that can modulate and influence brain activity, therefore representing a promising diagnostic and therapeutic strategy for various neurological conditions [25]. The effects of rTMS on brain activity depend on its frequency: low-frequency rTMS lowers excitability, while high-frequency rTMS increases it. In clinical settings, low-frequency rTMS is used to inhibit activity in healthy brain structures, while high-frequency rTMS is usually applied to the affected brain region, to increase its activity. The specific brain regions targeted by rTMS depend on the type of dysfunction being treated, and the biological mechanisms potentially mediating clinical benefits remain to be clarified [26]. Preclinical studies in animal models have shown that rTMS reduces edema and modulates the proliferation and differentiation of neural stem cells (NCS) via the regulation of Ca++, CREB, and BDNF pathways and miRNA [27]. rTMS might also be associated with the improvement of the glymphatic system’s drainage function and brain parenchymal metabolite clearance after ICH in mice [28].

### 3.2. Transcranial Direct Current Stimulation (tCDS)

tDCS works by delivering an electrical current (1–2 mA) through electrodes placed on the scalp. The impact on brain activity varies depending on the current direction: the anode (positive electrode) increases excitability by depolarizing the membranes’ potential, while the cathode decreases excitability by hyperpolarizing it. The effects of tDCS are not limited to the stimulated area and can also influence nearby brain regions, potentially modulating and reshaping the connections across broader brain networks [29]. A recent study confirmed that tCDS enhances BDNF levels after stroke, consequently increasing cerebral structural plasticity and connectivity [30]. Increased BDNF levels might therefore represent the biological mediator linking tDCS with improved connectivity after stroke.

### 3.3. Transcranial Alternating Current Stimulation (tACS)

Transcranial Alternating Current Stimulation (tACS) works through the application of a low-intensity alternating electrical current through electrodes placed on the scalp. The aim of this stimulation is to modulate brain waves and synchronize the activity of neurons [31,32]. By adjusting the current’s frequency, tACS can either synchronize or desynchronize brain oscillations, leading to specific effects on several cognitive functions and attention [33,34].

Research based on animal models and computational models suggests that tACS works by influencing the brain’s natural electric fields, therefore influencing the activity of neural networks [35,36]. The impact of tACS interacts with the brain’s spontaneous activity state during the stimulation, and its effects can last beyond the stimulation period [37].

### 3.4. Non-Invasive Vagus Nerve Stimulation (nVNS)

Non-invasive Vagus Nerve Stimulation (nVNS) delivers mild electrical impulses to the vagus nerve, a key component of the autonomic nervous system that regulates different functions like heart rate, inflammation, and pain perception [38]. Unlike invasive methods, nVNS uses skins electrodes, usually on the neck or in the outer ear, to stimulate the afferent fibers of the vagus nerve [39,40]. The stimulation of the vagus nerve triggers activity in key areas of the brain, such as the nucleus tractus solitarius in the brainstem. This neural activity can then affect higher-order regions like the insula and prefrontal cortex, areas involved in pain processing, emotional regulation, and cognitive functions [38].

nVNS has also been shown to modulate brain oscillations, particularly in gamma and theta frequencies, which are associated with attention, pain, and emotional processing [41]. Overall, nVNS represents a targeted neurostimulation approach that leverages the vagus nerve’s extensive influence on the central and peripheral nervous systems to modulate brain activity, improve autonomic balance, and modulate a variety of neurological and systemic disorders [38].

### 3.5. NIBS to Predict ICH Outcome

A total of five studies investigating the prognostic value of NIBS, all of which used TMS, were identified. As shown in Table 3, TMS can be used to assess corticospinal tract (CST) damage by measuring motor-evoked potentials (MEPs).

Reduced MEP amplitudes, increased latency, or MEP absence is associated with poor functional outcome, while normal MEPs are a reliable indicator of anatomical continuity and normal functioning of the corticospinal motor pathways, which are associated with a higher likelihood of motor recovery and favorable prognosis. Patients with no response after cortical stimulation had higher mortality and poor outcomes.

TMS timing appears crucial to predict ICH prognosis, and 14 days after the index event seems the best time to estimate functional outcome and recovery. TMS might be a reliable alternative to DTT on MRI to evaluate the residual function of corticospinal fibers, although these two techniques have different diagnostic performance, namely a higher positive predictive value in the former and a better negative predictive value in the latter.

Table 3 summarizes the available evidence on the prognostic value of NIBS.

**Table 3:** NIBS to predict ICH outcome.

Study	Study Design	Intervention	Stimulation	Timing	Outcome Measures	Main Findings
Jang, et al., 2007 [12]	Case report	TMS in the evaluation of CST status	ET defined as the minimum stimulus required to elicit an MEP with a peak-to-peak amplitude of 50 KV or greater in 2 of 4 attempts. Stimulation intensity was set at the ET plus 20% of maximum stimulator output. One hemisphere was stimulated 4 times at a minimum of 10 s intervals.	2 weeks and 14 weeks after onset	MEP comparison from both abductor pollis	TMS might reflect CST status
Fekete et al., 2021 [13]	Observational	TMS and EEG in the evaluation of CST status	Abductor digiti minimi muscle on both upper limbs and both tibial anterior muscles on the lower limbs’ MEP measurement. A 20% above-threshold and maximal stimulation output on cortical cervical and lumbar regions. Four stimulations.	24 to 48 h after admission, at 14 days ± 2 days and 3 months ± 7 days	Case fatality at discharge, 3-month mortality, functional outcome at 3 months ± 7 days after the onset	MEP by TMS is a useful early prognostic marker; evaluation at 14 days is best TMS timing for prognostication
Jang et al., 2010 [13,14]	Observational	Comparison of TMS vs. DTT in the evaluation of CST status	MEPs obtained from both abductor pollicis brevis muscles (APBs) in a relaxed state. The excitatory threshold (ET) was defined as the minimum stimulus required to elicit an MEP with a peak to peak amplitude of 50 μV or greater in two of four attempts. Stimulation intensity was set at the ET plus 20% of the maximum stimulator output. One hemisphere was stimulated four times at a minimum of 10 s intervals.	TMS within 7–28 days after onset	Modified Brunnstrom classification and motricity index of upper extremity (UMI) evaluation at onset and at 6 months	TMS had higher positive predictive value while DTT had higher negative predictive value in predicting motor outcome
Nagao and Kawai, 1992 [15]	Observational	TMS	Capacitors charged to a maximum output of 1 kV were discharged through the coil. The magnetic field, which approached 2 Tesla at the coil center at maximum output, had a peak at about 150 sec. Surface electrodes recorded the compound muscle action potential (MEP) elicited in the thenar muscles of both the affected and normal hands.	MEP examination was performed at approximately 1 week and 1, 2, and 3 months	Manual motor test (MMT), range 0–5	Good correlation between MEPS suppression and motor functional outcome
Shah and Kalita et al., 2005 [16]	Observational	TMS	MEP recording from abductor digiti minimi. Median somatosensory evoked potentials (SEP) were obtained by stimulating the median nerve at the wrist and recording from Erb’s point and the parietal cortex	MEP evaluation within 6 days from onset	Barthel Index	MEP and SEP abnormality are significant predictors of outcome

TMS: Transcranial magnetic stimulation; MEP: motor-evoked potentials; SEP: somatosensory-evoked potentials, ET: excitatory threshold; DTT: diffusion tensor tractography; CST: corticospinal tract; ICH: intracerebral hemorrhage, SD standard deviation; GCS: Glasgow Coma Scale.

### 3.6. NIBS to Enhance Functional Recovery

Table 4 summarizes the available evidence on NIBS to facilitate functional recovery in ICH survivors. We observed significant heterogeneity in the technical approach, and rTMS was the more commonly used stimulation method. We also observed that different outcomes of interest were studied, varying from swallowing function to motor function, ataxia, ADL, and mRS.

Overall, NIBS seems a promising approach to promote functional recovery, especially when coupled with occupational and physical therapy [21,23]. The observed quality of evidence was higher compared to prognostication studies, with robust data from randomized studies. However, the interpretation of available evidence remains challenging because of significant heterogeneity in the characteristics of included patients, stimulation protocols, and main outcome of interest. We also observed significant heterogeneity in the timing of the stimulation and functional outcome assessment, preventing a clear comparison between different techniques and different studies.

## 4. Discussion

Overall, NIBS appears to be a safe and promising approach to enhance functional recovery after ICH and stratify the risk of poor outcome in ICH patients. The majority of available studies used TMS, and there is great heterogeneity in brain stimulation timing and outcomes of interest. Conversely, there is very limited evidence (single case report) of the safety and efficacy of VNS in ICH patients [42].

tDCS seems the more promising method to improve upper limb motor function, particularly in patients with cortical or subcortical ICH, although improvement in ADL has not been demonstrated so far. Compared to rTMS, tDCS offers several advantages, including ease of use, portability, and better patient tolerance. It can be easily administered at home by occupational and physiotherapists in primary care settings, is well tolerated by patients, and is suitable for use in blinded clinical trials [26,43].

No major adverse effects were described, and there is no evidence of an increased risk of adverse events such as seizure, neurological deterioration, or rebleeding. tCDS’s most common adverse effects were transient itching, tingling sensations at the electrodes site, mild headache, and general discomfort.

Our analysis of the role of NIBS in promoting functional recovery and improving prognostication after ICH highlighted several limitations and unmet needs that have probably reduced reproducibility and hindered a widespread adoption of NIBS in clinical practice.

First, the majority of available evidence in the literature was obtained in patients with ischemic stroke, whereas ICH remains underrepresented in NIBS studies, with small sample sizes that have prevented statistically powered analyses.

Second, there is great heterogeneity in NIBS timing, stimulation techniques, outcomes of interest, and adjustment for major confounders. Inconsistency in selection criteria and treatment protocols has probably reduced the reproducibility of several previous findings. Furthermore, variability in patients’ selection has prevented a direct comparison between different techniques and protocols. Third, the individualization of NIBS following a precision medicine approach has been challenging, mainly because of limited sample sizes and reduced evidence on patients’ subgroups more likely to derive clinical benefit.

Third, few studies accounted for known prognostic factors, and it remains unclear whether NIBS can improve the discriminative ability of currently available, validated prognostic scores. Fourth, it appears biologically plausible that NIBS has different impacts and values based on ICH location (i.e., deep versus lobar supratentorial ICH) and underlying small vessel disease, and further studies are needed to test this hypothesis. As shown in Table 2, the patients examined presented primarily deep ICH. Lobar ICHs could likely under-select due to specific exclusion, including intact higher cognitive functions and only motor symptom presentation.

Fifth, the ICH recovery trajectory continues up to one year after the index event and most of the available studies assessed outcome at a very early stage of the disease [3]. Sixth, the interaction between NIBS and traditional physical therapy remains unanswered. Seventh, it remains unclear whether a subset of patients are more likely to benefit from NIBS, representing therefore the ideal candidates for future randomized trials. Eight, although improved brain plasticity and increased BDNF levels appear plausible biological mediators of the effects of NIBS, the precise pathophysiological mechanisms underlying the effect of NIBS remain poorly characterized. Ninth, regulatory and training barriers, limited accessibility, and resource availability appear to be major determinants that explain the lack of widespread implementation of NIBS in clinical practice and research.

Finally, it is difficult to quantify the real added value of NIBS to currently available prognostication and recovery tools, and the most promising approach appears to be the integration of NIBS into the multidisciplinary care of ICH survivors.

## 5. Conclusions

NIBS appears to be a valuable approach to promote recovery and estimate prognosis in ICH survivors. Heterogeneity in stimulation protocols, study populations, and outcomes of interest prevent the implementation of NIBS in daily clinical practice. Further research is needed to prospectively validate the available promising evidence.

## Figures and Tables

**Table 1:** Main Predictors of ICH outcome.

Age	Older age is a strong predictor of mortality and poor recovery after ICH. Age-related comorbidities and reduced brain resilience contribute to these outcomes.
Baseline mRS	Pre-existing disability may complicate acute management and rehabilitation. Patients with worse baseline function may be less likely to tolerate complications, engage in rehabilitation, or return to pre-ICH functional levels
GCS	The GCS score on admission correlates with outcomes. A lower GCS score (≤8) predicts higher mortality and worse functional outcomes [6]. Higher GCS is independently associated with reduced risk of neurological deterioration [5].
ICH volume	Larger hematoma volumes are associated with worse outcomes, including higher mortality and severe disability rates [7]. The volume cut off for predicting neurological outcomes depends on ICH location [8].
Hematoma expansion	Hematoma expansion (volume increase >33% and or >6 mL) is a major determinant of neurological deterioration and poor prognosis [9,10]. Patients on antiplatelet or anticoagulant treatment have worse outcomes due to an increased risk of hematoma expansion.
ICH location and presence of IVH	Some studies reported better outcomes in lobar ICH [11], while other studies have shown no significant mRS differences between deep and lobar ICH at all time points (mRS 3–12 months) [5]. Infratentorial location and IVH presence are known predictors of unfavorable functional outcome.

ICH: intracerebral hemorrhage; mRS: modified Rankin scale; GCS: Glasgow coma scale; IVH: intraventricular hemorrhage.

**Table 2:** Characteristics of included studies.

Study	Sample Size (% Female)	Mean Age + SD (Years)	ICH Characteristics	Admission National Institute of Heath Stroke Scale (NIHSS)	Admission Glasgow Coma Scale (GCS)	Admission Modified Rankin Scale (mRS)	Other Evalutation Scale	Patient Selection and Main Inclusion/Exclusion Criteria
Jang, et al., 2007 [12]	1 F	32	Lobar location	n.a (complete paralysis of left limbs)	n.a	n.a	No	Single-patient case report
Fekete et al., 2021 [13]	74 (35.5%)	70 ± 11	75% deep ICH; mean ICH volume 26 mL	14.25 (IQR 8–19.25)	12 ± 3	9 (7.7%) mRs 0–2 (among 116 patients enrolled)	No	Exclusion of vascular malformations, trauma, malignancy and other causes of secondary ICH
Jang et al., 2010 [13,14]	53 (41.5 %)	54 ± 10	ICH involving the CST at the corona radiata or posterior limb of internal capsule level	n.a. (severe weakness of the affected limbs)	n.a	n.a	All had a modified Brunnstrom classification (MBC) of 0 and mean motricity index of upper extremity (UMI) 3.94 ± 8.16	Exclusion of patients with apraxia, somatosensory problems, or cognitive impairment (Mini Mental State Examination <25/30)
Nagao and Kawai, 1992 [15]	13 (46%)	57.8	11 deep location (84.6%)	n.a	n.a	n.a	76.9% patients showed a Manual Motor Test of 0 (no strength)	n.a.
Shah and Kalita et al., 2005 [16]	53 (34%)	58.8	Thalamic ICH; 9.5% < 2 cm [17] 66% 2–4 cm 24.5% >4 cm	n.a	10.4	n.a.	Canadian Neurological Scale (CNS), mean 3.9	Patients with CT-proven thalamic hemorrhage referred within 6 days from onset
Mortensen et al., 2015 [18]	16 (43%)	n.a	3 posterior fossa and midbrain, 11 deep, 2 lobar	n.a	n.a	n.a	n.a.	Inclusion criteria: age 18–80 years, >6 months and <5 years from the initial ICH; Exclusion criteria: trauma, epilepsy, metal cranial devices, other neurological diseases, cognitive impairment
Fujiki et al., 2022 [19]	55 (9%)	58.5 ± 10.8	18 deep ICH (32% of the total); >5 and <30 mL in volume	n.a	n.a	n.a	Modified water swallowing test (MWST)	ICH volume 5–30 mL, no surgical therapy, no neurological deficits apart from swallowing and motor dysfunction
Jiaqia Ke, Jiana Wei et al., 2022 [20]	26 (46%)	Intervention group 58 (46.5–63); sham group 56 (46.5–61.5)	All basal ganglia/thalamus ICH	mean rTMS group NIHSS 9 ± 3 SD, mean sham group NIHSS 7.7 ± 4.4 SD	n.a	n.a	Fugl–Meyer Assessment (FMA) and Medical Research Council (MRC) scale	Age 18–70 years, within 8 weeks of first-ever ICH; Exclusion criteria: traumatic ICH, other; Neurological disorders, arrhythmia, fever, infection and epilepsy; severe aphasia or cognitive impairment
Tatsuno et al., 2021 [21]	840 ICH, (34%F)	60 (54–68)	n.a	n.a	n.a	n.a	Fugl–Meyer assessment (FMA) test	Inclusion criteria: upper limb paresis associated with ICH, age > 20 years, ≥6 months since the stroke, history of a single stroke; Exclusion criteria: cognitive disabilities (MMSE lower than 26), active physical or mental illness requiring medical management, history of seizures for ≥1 year, intracranial metal clips or intracardiac pacemaker, chemodenervation of the affected upper limb with phenol or botulinum toxin, and subarachnoid hemorrhage
Urishidani et al., 2017 [22]	7 (57%)	67.7	All thalamic ICH	n.a	n.a	n.a	FMA, Fugl–Meyer assessment; ICARS, International Cooperative Ataxia Rating scale; MAS, modified Ashworth scale	Inclusion criteria: Brunnstrom recovery stage 4–6 for the hands and fingers, and the upper limb of the affected side; a cerebellar type of discoordination, no sensory deficit; no evidence of a visual field defect; age at intervention 18–80 years; time after onset of stroke of more than 12 months; history of a single symptomatic stroke only (no bilateral cerebrovascular lesion); no cognitive impairment with a pre-treatment Mini-Mental State Examination score of more than 24; no active physical or mental illness requiring medical management; no recent history of seizure
Komatsu et al., 2022 [23]	44 (32%)	56 (48–63)	37 (84%) deep ICH, 7 (16%) brain stem ICH; median ICH volume 10 mL (4.3–18)	12 (IQR 9–18)	14 (IQR 13–15)	n.a	no	Inclusion criteria: paralysis with an NIHSS score of 1 or higher for at least 3 days after onset; Exclusion criteria: cortical, subcortical, and cerebellar ICH, intraventricular hemorrhage, history of symptomatic stroke, surgical management for ICH, impaired consciousness, age over 80 and seizures

Values presented are mean ± standard deviation SD (range) or median [IQR]. n.a = not available.

**Table 4:** NIBS to enhance functional recovery.

Study	Study Design	Intervention	Stimulation	Timing	Outcome Measures	Main Findings
Mortensen et al., 2015 [18]	Double-blind sham-controlled RCT	tCDS	Anodal tDCS consisted of 20 min of stimulation with a 30 s fade in/fade out sequence. The current was delivered at 1.5 mA, which gave a current density of 0.04 mA/cm^2^. Sham tDCS consisted of a 30 s fade in/fade out sequence at the beginning of the session.	Randomisation: five consecutive days of occupational therapy combined with either anodal or sham tDCS	ADL performance with Jebsen–Taylor test (JTT)	Added value of NIBS compared to occuaptional therapy alone: no improvement in ADL
Fujiki et al., 2022 [19]	Observational	TMS (QBS5)	Mylohyoid-MEPs after left motor cortical QBS5 (four sets of four monophasic pulses at frequency of 500 Hz, repeated at 200 Hz, i.e., 5 ms interburst interval, with an inter-train interval of 5 s) conditioning.	Evaluation at 1–7 median days after onset	Modified water swallowing test (MWST)	QBS5 robust facilitation in the bilateral mylohyoid MEPs
Jiaqia Ke, Jiana Wei et al., 2022 [20]	Double-blind sham-controlled RCT	HF-rPMS	rPMS was applied at a frequency of 20 Hz and delivered for 1 sec, with an interval of 19 sec between stimuli. In total, 1800 pulses were delivered (a period of 30 min stimulation per day) at each stimulation site. The intensity was individually set to 40–60% output intensity of the stimulator, which evoked significant movement of the affected limbs, avoiding uncomfortable responses. Coils were placed at the armpit and the popliteal fossa.	Within 8 weeks from ICH onset	Fugl–Meyer Assessment (FMA) scale and Medical Research Council (MRC) scale before and after HF-rPMS	Synchrous HF-rPMS improved motor function and proximal muscle strength of upper and lower limb
Tatsuno et al., 2021 [21]	Observational	rTMS	Patient received 40 min low-frequency rTMS therapy and 60 min occupational therapy twice daily, 6 days/week; 2400 stimuli of 1 Hz each were applied for 40 min to the contralateral hemisphere over the primary motor area. The stimulation intensity was set at 90% of the resting motor threshold of the first dorsal interosseous muscle.	15 days of hospitalization to receive rTMS and occupational therapy	Upper Fugl–Meyer assessment (FMA)	Long-term upper extremity muscle paralysis can be improved by NEURO equally in patients with CI and ICH
Urishidani et al., 2017 [22]	Observational	rTMS	20 min rTMS at 1 Hz (LF-rTMS) hemisphere on the healthy hemisphere followed by 120 min intensive occupational therapy, daily for 21 sessions (NEURO 15 protocol). rTMS of 1200 pulses at 1 Hz was applied to the skull of the nonlesional hemisphere at a site that elicited the largest motor- evoked potentials in the first dorsal interosseous muscle of the unaffected upper limb on surface electromyography.	After 12 months from onset	Fugl-Meyer Assessment (FMA) And International Cooperative Ataxia Rating Scale (ICARS)	NEURO-15 intervention may be beneficial not only for upper-limb hemiparesis but also for ipsilateral ataxia
Komatsu et al., 2022 [23]	Observational	HF-rTMS in acute ICH lesioned side	10 s trains of 10 Hz were applied repeatedly with 20 s inter-train intervals over 12 min (a total of 2400 pulses per session). The optimal site of stimulation was defined as the location where the largest motor-evoked potentials (MEPs) in the first dorsal interosseous muscle or tibialis anterior muscle of the paralyzed upper or lower limb was elicited on a surface electromyograph. The HF-rTMS intensity was 90–100% of the resting motor threshold at the stimulation site.	Patients received rTMS for 10 days within 2 weeks in parallel with conventional rehabilitation	Favorable outcome mRs (0–2) at 3 months (HF-rTMS + OT vs. OT alone)	HF-rTMS combined with conventional rehabilitation was independently associated with favorable outcome at 3 months

TMS: Transcranial magnetic stimulation; rTMS repetitive transcranial magnetic stimulation; HF-rTMS: high-frequency repetitive transcranial magnetic stimulation; HF-rPMS: high-frequency repetitive peripheral magnetic stimulation; QBS: quadri-burst stimulation; MEP: motor-evoked potentials; ICH: intracerebral hemorrhage; GCS: Glasgow coma scale; OT: occupational therapy; NIHSS: National Institutes of Health Stroke Scale.

## References

[1] Puy L., Parry-Jones A.R., Sandset E.C., Dowlatshahi D., Ziai W., Cordonnier C. (2023). Intracerebral haemorrhage. Nat. Rev. Dis. Primers.

[2] Feign V.L., Forouzanfan M.H., Krishnamurthi R., Mensah G.A., Connor M., Bennett B.A., Moran A.E., Sacco R.L., Anderson L., Truelsen T. (2014). Global and regional burden of stroke during 1990–2010. Lancet.

[3] Witsch J., Siegerink B., Nolte C.H., Sprügel M., Steiner T., Endres M., Huttner H.B. (2021). Prognostication after intracerebral hemorrhage: A review. Neurol. Res. Pract..

[4] Shah V.A., Thompson R.E., Yenokyan G., Acosta J.N., Avadhani R., Dlugash R., McBee N., Li Y., Hansen B.M., Ullman N. (2022). One-year outcome trajectories and factors associated with functional recovery among survivors of intracerebral and intraventricular hemorrhage with initial severe disability. JAMA Neurol..

[5] Morotti A., Nawabi J., Pilotto A., Toffali M., Busto G., Mazzacane F., Cavallini A., Laudisi M., Gentile L., Viola M.M. (2024). Functional outcome improvement from 3 to 12 months after intracerebral hemorrhage. Eur. Stroke J..

[6] Hemphill J.C.I.I.I., Bonovich D.C., Besmertis L., Manley G.T., Claiborne Johnston S. (2001). The ICH Score A Simple, Reliable Grading Scale for Intracerebral Hemorrhage. Stroke.

[7] Cordonnier C., Demchuk A., Ziai W., Anderson C.S. (2018). Intracerebral haemorrhage: Current approaches to acute management. Lancet.

[8] Teo K.C., Fong S.M., Leung W.C., Leung I.Y., Wong Y.K., Choi O.M., Yam K.K., Lo R.C., Cheung R.T., Ho S.L. (2023). Location-Specific Hematoma Volume Cutoff and Clinical Outcomes in Intracerebral Hemorrhage. Stroke.

[9] Dowlatshahi D., Demchuk A.M., Flaherty M.L., Ali M., Lyden P.L., Smith E.E. (2011). Defining hematoma expansion in intracerebral hemorrhage: Relationship with patient outcomes. Neurology.

[10] Salman R.A., Frantzias J., Lee R.J., Lyden P.D., Battey T.W., Ayres A.M., Goldstein J.N., Mayer S.A., Steiner T., Wang X. (2018). Absolute risk and predictors of the growth of acute spontaneous intracerebral haemorrhage: A systematic review and meta-analysis of individual patient data. Lancet Neurol..

[11] Rost N.S., Smith E.E., Chang Y., Snider R.W., Chanderraj R., Schwab K., FitzMaurice E., Wendell L., Goldstein J.N., Greenberg S.M. (2008). Prediction of functional outcome in patients with primary intracerebral hemorrhage: The FUNC score. Stroke.

[12] Jang S.H., Ahn Y.H., Kim S.H., Chang C.H. (2007). Corticospinal Tract Restoration: Combined Study of Diffusion Tensor Tractography, Functional MRI, and Transcranial Magnetic Stimulation. J. Comput. Assist. Tomogr..

[13] Fekete K., Tóth J., Horváth L., Márton S., Héja M., Csiba L., Árokszállási T., Bagoly Z., Sulina D., Fekete I. (2021). Neurophysiological Examinations as Adjunctive Tool to Imaging Techniques in Spontaneous Intracerebral Hemorrhage: IRONHEART Study. Front. Neurol..

[14] Jang S.H., Ahn S.H., Sakong J., Byun W.M., Choi B.Y., Chang C.H., Bai D., Son S.M. (2010). Comparison of TMS and DTT for predicting motor outcome in intracerebral hemorrhage. J. Neurol. Sci..

[15] Nagao S., Kawai N. (1992). Prediction of Motor Function by Magnetic Brain Stimulation in Patients with Intracerebral Hematoma. Neurol. Med. Chir..

[16] Shah S.D., Kalita J., Misra U.K., Mandal S.K., Srivastava M. (2005). Prognostic predictors of thalamic hemorrhage. J. Clin. Neurosci..

[17] Caplan L.R. (1992). Intracerebral haemorrhage. Lancet.

[18] Mortensen J., Figlewski K., Andersen H. (2016). Combined transcranial direct current stimulation and home-based occupational therapy for upper limb motor impairment following intracerebral hemorrhage: A double-blind randomized controlled trial. Disabil. Rehabil..

[19] Fujiki M., Hata N., Anan M., Matsushita W., Kawasaki Y., Fudaba H. (2023). Monophasic-quadri-burst stimulation robustly activates bilateral swallowing motor cortices. Front. Neurosci..

[20] Ke J., Wei J., Zheng B., Tan T., Zhou W., Zou X., Zou H., Zeng H., Zhou G., Chen L. (2022). Effect of High-Frequency Repetitive Peripheral Magnetic Stimulation on Motor Performance in Intracerebral Haemorrhage: A Clinical Trial. J. Stroke Cerebrovasc. Dis..

[21] Tatsuno H., Hamaguchi T., Sasanuma J., Kakita K., Okamoto T., Shimizu M., Nakaya N., Abo M. (2021). Does a combination treatment of repetitive transcranial magnetic stimulation and occupational therapy improve upper limb muscle paralysis equally in patients with chronic stroke caused by cerebral hemorrhage and infarction? A retrospective cohort study. Medicine.

[22] Urushidani N., Okamoto T., Kinoshita S., Yamane S., Tamashiro H., Kakuda W., Abo M. (2017). Combination Treatment of Low-Frequency Repetitive Transcranial Magnetic Stimulation and Intensive Occupational Therapy for Ataxic Hemiparesis due to Thalamic Hemorrhage. Case Rep. Neurol..

[23] Komatsu T., Hada T., Sasaki N., Kida H., Takahashi J., Maku T., Nakada R., Shiraishi T., Akiyama S., Kitagawa T. (2022). Effects and safety of high-frequency rTMS in acute intracerebral hemorrhage patients: A pilot study. J. Neurol. Sci..

[24] Hallett M. (2007). Transcranial Magnetic Stimulation: A Primer. Neuron.

[25] Rossini P.M., Rossi S. (2007). Transcranial magnetic stimulation: Diagnostic, therapeutic, and research potential. Neurology.

[26] George M.S., Aston-Jones G. (2009). Noninvasive techniques for probing neurocircuitry and treating illness: Vagus nerve stimulation (VNS), transcranial magnetic stimulation (TMS) and transcranial direct current stimulation (tDCS). Neuropsychopharmacology.

[27] Cui M., Ge H., Zeng H., Yan H., Zhang L., Feng H., Chen Y. (2019). Repetitive Transcranial Magnetic Stimulation Promotes Neural Stem Cell Proliferation and Differentiation after intracerebral Hemorrhage in Mice. Cell Transplant..

[28] Liu Y., Liu X., Sun P., Li J., Nie M., Gong J., He A., Zhao M., Yang C., Wang Z. (2023). rTMS treatment for abrogating intracerebral hemorrhage-induced brain parenchymal metabolite clearance dysfunction in male mice by regulating intracranial lymphatic dreinage. Brain Behav..

[29] Nitsche M.A., Paulus W. (2011). Transcranial direct current stimulation—Update 2011. Restor. Neurol. Neurosci..

[30] Longo V., Barbati S.A., Re A., Paciello F., Bolla M., Rinaudo M., Miraglia F., Alù F., Di Donna M.G., Vecchio F. (2022). Transcranial Direct Current Stimulation Enhances Neuroplasticity and Accelerates Motor Recovery in a Stroke Mouse Model. Stroke.

[31] Antal A., Paulus W. (2013). Transcranial alternating current stimulation (tACS). Front. Hum. Neurosci..

[32] Herrmann C.S., Rach S., Neuling T., Strüber D. (2013). Transcranial alternating current stimulation: A review of the underlying mechanisms and modulation of cognitive processes. Front. Hum. Neurosci..

[33] Vosskuhl J., Strüber D., Herrmann C.S. (2018). Non-invasive Brain Stimulation: A Paradigm Shift in Understanding Brain Oscillations. Front. Hum. Neurosci..

[34] Witkowski M., Garcia-Cossio E., Chander B.S., Braun C., Birbaumer N., Robinson S.E., Soekadar S.R. (2016). Mapping entrained brain oscillations during transcranial alternating current stimulation (tACS). NeuroImage.

[35] Fröhlich F., McCormick D.A. (2010). Endogenous Electric Fields May Guide Neocortical Network Activity. Neuron.

[36] Reato D., Rahman A., Bikson M., Parra L.C. (2013). Effects of weak transcranial alternating current stimulation on brain activity—A review of known mechanisms from animal studies. Front. Hum. Neurosci..

[37] Neuling T., Rach S., Herrmann C.S. (2013). Orchestrating neuronal networks: Sustained after-effects of transcranial alternating current stimulation depend upon brain states. Front. Hum. Neurosci..

[38] Yuan H., Silberstein S.D. (2016). Vagus Nerve and Vagus Nerve Stimulation, a Comprehensive Review: Part I. Headache J. Head Face Pain.

[39] Frangos E., Ellrich J., Komisaruk B.R. (2014). Non-invasive Access to the Vagus Nerve Central Projections via Electrical Stimulation of the External Ear: fMRI Evidence in Humans. Brain Stimul..

[40] Dietrich S., Smith J., Scherzinger C., Hofmann-Preiß K., Freitag T., Eisenkolb A., Ringler R. (2008). A novel transcutaneous vagus nerve stimulation leads to brainstem and cerebral activations measured by functional MRI. Biol. Psychiatry.

[41] Busch V., Zeman F., Heckel A., Menne F., Ellrich J., Eichhammer P. (2013). The effect of transcutaneous vagus nerve stimulation on pain perception—An experimental study. Brain Stimul..

[42] Cummins D.D., Kalagara R., Downes M.H., Park H.J., Tosto-Mancuso J., Putrino D., E Panov F., Kellner C.P. (2024). Vagus nerve stimulation for enhanced stroke recovery after intracerebral hemorrhage: Illustrative case. J. Neurosurg. Case Lessons.

[43] Gandiga P.C., Hummel F.C., Cohen L.G. (2006). Transcranial DC stimulation (tDCS): A tool for double-blind sham-controlled clinical studies in brain stimulation. Clin. Neurophysiol..

